# RNAi-Mediated *FoxO* Silencing Inhibits Reproduction in *Locusta migratoria*

**DOI:** 10.3390/insects15110891

**Published:** 2024-11-14

**Authors:** Jiaying Xu, Zeming Yuan, Huazhang Zhao, Xinru Wu, Nina Cai, Tingting Ma, Bin Tang, Gongxing Chen, Shigui Wang

**Affiliations:** 1 College of Life and Environmental Sciences, Hangzhou Normal University, Hangzhou 311121, China; 2023111010043@stu.hznu.edu.cn (J.X.); 2020210315046@stu.hznu.edu.cn (Z.Y.); 2022210301085@stu.hznu.edu.cn (H.Z.); 2020210315056@stu.hznu.edu.cn (X.W.); 2020210315057@stu.hznu.edu.cn (N.C.); 2022111010007@stu.hznu.edu.cn (T.M.); tbzm611@163.com (B.T.); 2 School of Pharmacy, Hangzhou Normal University, Hangzhou 311121, China

**Keywords:** *Locusta migratoria*, RNAi, *FoxO*, Hippo pathway, reproduction

## Abstract

*FoxO* is a downstream target gene of cellular nutrient and growth factors, oxidative stress responses, and insulin signaling pathways. It play a crucial role in insect growth, development, and reproduction. *Locusta migratoria* is a significant agricultural pest; therefore, the identification of novel control targets for its management is of significant importance. After injecting dsRNA to interfere with *FoxO* expression, we observed changes in the reproduction-related gene expression and ovary development through RT-qPCR and morphological observation. Simultaneously, the trehalose and glycogen contents were measured following RNAi. The results demonstrate that interference with *FoxO* significantly downregulates key genes in the Hippo pathway and *Notch* gene expression. In terms of carbohydrate metabolism, the trehalose content decreases significantly while the glycogen content increases markedly after *FoxO* silencing. Additionally, *FoxO* silencing considerably inhibits reproductive-related gene expression, resulting in delayed ovarian development. These findings indicate that *FoxO* regulates *L. migratoria* reproduction through the Hippo signaling pathway: when impaired, the reproductive capacity function declines. In addition, *FoxO*-mediated energy mobilization is involved in the regulation of egg production. These results indicate that the RNAi of *FoxO* may be a useful control strategy against *L. migratoria*.

## Simple Summary

Locusts are significant agricultural pests; therefore, the identification of novel control targets for their management is of immense importance. *FoxO*, a downstream target gene of cellular nutrient and growth factors, oxidative stress responses, and insulin signaling pathways, plays a pivotal role in the growth, development, and reproduction of insects. *FoxO* silencing resulted in significant changes in the expressions of genes associated with reproduction and the Hippo pathway and significantly reduced ovary development. These findings indicate that *FoxO* regulates reproduction in *L. migratoria* through the Hippo signaling pathway: when impaired, the reproductive capacity function declines. In addition, *FoxO*-mediated energy mobilization is involved in the regulation of egg production. Overall, these results highlight the potential of targeting *FoxO* as a novel molecular approach for controlling *L. migratoria*.

## 1. Introduction

Reproduction is a crucial factor that influences the adaptability of insects [1]. In terms of insect reproduction, the occurrence of yolk directly impacts their reproductive capacity [2]. Yolk occurrence primarily involves vitellogenin (Vg) production in the fat body, its release into the hemolymph, and its uptake by mature oocytes [3,4]. In *Locusta migratoria*, developing oocytes selectively incorporate Vg from outside the egg through endocytosis mediated by the vitellogenin receptor (VgR) [5]. Once inside the oocyte, Vg is stored as crystalline vitellin, serving as a nutritional reserve for future embryonic development [6,7]. At the oogenesis stage, the Notch pathway is involved in the spatial and temporal regulation of follicle cell differentiation and proliferation [8,9]. In *L. migratoria*, the increase in JH expression ensures high *Notch* abundance, consequently contributing to successful egg production [10].

In addition, the insulin signaling pathway in insects can influence their reproduction by regulating Vg protein synthesis [4,11]. As downstream target genes for cellular nutrients, growth factors, oxidative stress responses, and insulin signaling pathways (IIS), *FoxO* exerts both activating and inhibitory functions through transcriptional regulation mediated by interactions with regulators [12,13]. It binds to multiple target gene promoters and further modulates physiological activities such as growth, development, and reproduction [14,15]. In insects, *FoxO* functions as a transcriptional repressor that binds to the promoter region of Vg. Upon phosphorylation, it is expelled from the cell nucleus, thereby triggering Vg synthesis [16,17]. *FoxO* exerts an impact on reproduction in various insects, including *Cyrtorhinus lividipennis*, *Tribolium castaneum*, and *Blattella germanica* [18,19,20]. In *B. germanica*, *FoxO* RNAi in fed females caused substantially reduced *Vg* expression and arrested oocyte growth [21]. Similarly, *FoxO* knockdown caused reductions in the *Vg* mRNA levels in fed *T. castaneum* adult females [22].

The Hippo signaling pathway is a cascade reaction that governs organ size by regulating cell growth, proliferation, and apoptosis. Additionally, it plays a pivotal role in stem cell renewal and tissue regeneration [23,24]. Its core constituents comprise Hippo (Hpo), Warts (Wts), and Yorkie (Yki), as well as the scaffold protein Salvador (Sav) [25,26,27,28]. Moreover, the Hippo pathway exerts essential control over the Notch receptor levels in follicle cells. The disruption of this pathway results in the aberrant differentiation of follicle cells, thereby impacting oocyte polarity [29,30]. In *Drosophila*, the Hippo pathway plays a crucial role in regulating follicle cell differentiation and oocyte polarity formation during ovarian development, in conjunction with the Notch, EGFR, and JAK-STAT pathways [30,31]. Both the EGFR and Hippo signaling pathways are indispensable for maintaining germ cell populations [32].

*L. migratoria* is a significant agricultural pest due to its short reproductive cycle, high reproduction rate, migratory behavior, and tendency to aggregate [33,34]. Therefore, the identification and exploration of novel locust control targets is of immense practical significance. In this study, we investigated the interplay between *FoxO* and the Hippo signaling pathway and elucidated the role of *FoxO* in regulating reproduction in *L. migratoria*. Our findings highlight the potential of targeting *FoxO* as a novel molecular approach for controlling *L. migratoria*.

## 2. Materials and Methods

### 2.1. Insects for Testing

Eggs of *L. migratoria* were purchased from a locust farm in Huaibei, Anhui Province. Locust eggs (50 g) were placed in a box (10 cm × 15 cm × 20 cm) with a layer of wet sand (2–3 cm) and reared at 30 ± 2 °C and 80% RH (relative humidity), with a 16 h light–8 h dark photoperiod. After hatching, the locusts were fed a mixture of fresh wheat seedlings and wheat bran. Approximately 200–300 individuals in each cage were placed in an insect cage (50 cm × 50 cm × 50 cm) in an artificial climate chamber. The feeding and temperature conditions were the same as those described above.

### 2.2. Bioinformatic Analysis of LmFoxO

The LmFoxO protein sequence (accession number QJX15634.1) was retrieved from GeneBank. The cDNA sequence of the *FoxO* gene was obtained from the locust transcriptomic database and was identified from genomic data on *L. migratoria* [35]. The ExPASy Proteomics website (http://web.expasy.org/protparam/ (accessed on 1 July 2021)) was used to predict the molecular mass and isoelectric point of LmFoxO. The SMART tool (http://smart.embl.de/ (accessed on 1 July 2021)) was used to predict the conserved structural domains of the FoxO protein. The BLAST search developed by the NCBI compared the homology of locusts with other species, selected the top 10 sequences with the highest identity, and used the multiple sequence results of MEGA 11 to build the evolutionary tree.

### 2.3. RNA Extraction and RT-qPCR

Total RNA was extracted using the Trizol reagent (TaKaRa, Dalian, China). The RNA concentration was determined using a NanoDrop 2000 spectrophotometer (Thermo Scientific, Waltham, MA, USA). Reverse transcription (RT) reactions were carried out using the PrimeScript RT Reagent Kit (Takara, Dalian, China). The cDNA was diluted 10 times for the subsequent general polymerase chain reaction (PCR), reverse transcription quantitative PCR (RT-qPCR), and dsRNA synthesis studies.

RT-qPCR was performed using a Bio-Rad Real-Time PCR Detection System (Bio-Rad, Hercules, CA, USA). All RT-PCR primers were designed using Primer 5.0 software (Table 1). *Lmβ-actin* was used as the internal reference gene. The gene expressions of *FoxO*, *Hpo*, *Sav*, *Yki*, *Met*, and *Vg* were detected via real-time fluorescence quantitative PCR using 10.0 μL of the PCR reaction system, 5 μL of SYBR Premix Ex Taq (Takara, Japan), 0.4 μL of forward primer, 0.4 μL of reverse primer, 1 μL of template cDNA, and 3.2 μL of RNase-free ddH_2_O. The reaction procedure included an initial pre-denaturation at 95 °C for 3 min, followed by 32 cycles of denaturation at 95 °C for 30 s, annealing at 58 °C for 30 s, and extension at 72 °C for 10 min. The relative expressions of the target genes were calculated using the 2^−△△CT^ method [36].

### 2.4. Tissue Expression Analysis of FoxO

To investigate the tissue-specific expression pattern of *FoxO*, five tissues were dissected from adult locusts (12 h post-adult eclosion): ovary, fat body, integument, midgut, and brain tissues. All samples were collected with three biological replicates, with five locusts per sample. The samples were snap-frozen in liquid nitrogen and stored at −80 °C for the subsequent total RNA extraction. The tissue-specific expression pattern of *FoxO* was analyzed using RT-qPCR.

### 2.5. RNAi-Mediated FoxO Silencing

To further investigate the function of FoxO, we employed RNA interference (RNAi) to knock down the expressions of target genes, with the green fluorescent protein (*GFP*) gene serving as a negative control. Due to the high GC content in the *FoxO* genome, nested PCR was chosen for its amplification. The specific primers used for the *FoxO* PCR amplification and dsRNA synthesis were designed using Primer 5.0 software (Table 1). The thermal profile for the nested PCR consisted of an initial denaturation at 95 °C for 5 min, followed by 30 cycles of denaturation at 95 °C for 30 s and annealing at 55 °C for 30 s. The ds*FoxO* and ds*GFP* were synthesized in vitro using the T7RiboMAX Express RNAi System (Promega Corporation, Madison, WI, USA), and they were purified following Tenlen’s method, described previously [37]. The synthesized ds*FoxO* and ds*GFP* were dissolved in ddH_2_O, and the final concentration was adjusted to 2 μg/μL. Approximately 20 μg (10 μL) of ds*FoxO* was injected into the abdomen between the second and third abdominal segments of each female locust in the early eclosion phase. All locusts treated with dsRNA were maintained under identical conditions, as described above, for the subsequent analysis. Samples were collected 5 days after injection for further analyses. The other parts of the locusts were bred until the insects died, and the weight of each pod was weighed and recorded. At the same time, the pod was carefully opened with a writing brush, and the number of eggs in each pod was counted.

### 2.6. Glycogen and Trehalose Determination

For the glycogen and trehalose content measurements, hemolymph was collected 5 days after injection for subsequent analysis. Each group included three biological replicates of five locusts. The samples were then centrifuged at 4 °C for 20 min at 3500 rpm to remove the hemocytes. Subsequently, 5 µL of hemolymph was mixed with PBS (32 µL) and 10% trichloroacetic acid (148 µL). The mixture was then centrifuged at 4 °C for 2 min at 10,000 rpm as the test sample.

The glucose standard curve was prepared with the glucose standard solution and the standard dilution with concentrations of 0 mg/L, 0.02 mg/L, 0.04 mg/L, 0.06 mg/L, 0.08 mg/L, and 0.1 mg/L. An amount of 30 µL of the sample was tested, the standard solution was taken, and 600 µL of the developer was added to a 90 °C water bath for 10 min, followed by an ice bath for 3 min. After mixing, the reaction mixture was added to the enzyme label plate, and the absorbance (A0) was determined at a wavelength of 620 nm.

The trehalose standard was diluted on a concentration gradient with preparations of 0.8 mM, 0.4 mM, 0.2 mM, 0.1 mM, and 0.05 mM as the standard curve test samples. An amount of 30 μL of the test samples or standard samples was added to a 1.5 mL Eppendorf (EP) tube, and 30 μL of 1% H_2_SO_4_ was added, followed by a 90 °C water bath for 10 min and an ice bath for 3 min. An amount of 30 μL of 30% KOH was added, followed by a 90 °C water bath for 10 min and an ice bath for 3 min. An amount of 600 μL of the developer (600 μL of 0.02 g of anthrone in 100 mL 80% H_2_SO_4_) was added, followed by a 90 °C water bath for 10 min and an ice bath for 3 min. After mixing, the reaction mixture was added to the enzyme label plate, and the absorbance was determined at a wavelength of 630 nm.

### 2.7. Data Statistics and Analysis

Data are expressed as means ± standard errors (SEs) and were evaluated for their normality and homogeneity of variance. Statistical analysis was performed using SPSS 26.0 software. One-way analysis of variance (ANOVA) followed by Tukey’s multiple range test was used to compare the differences between the treatment and control groups, and Student’s *t*-tests were used for the independent samples. All experiments were performed in triplicate with three biological replicates and at least three technical replicates.

## 3. Results

### 3.1. Bioinformatics Analysis of FoxO

**Figure 1:**
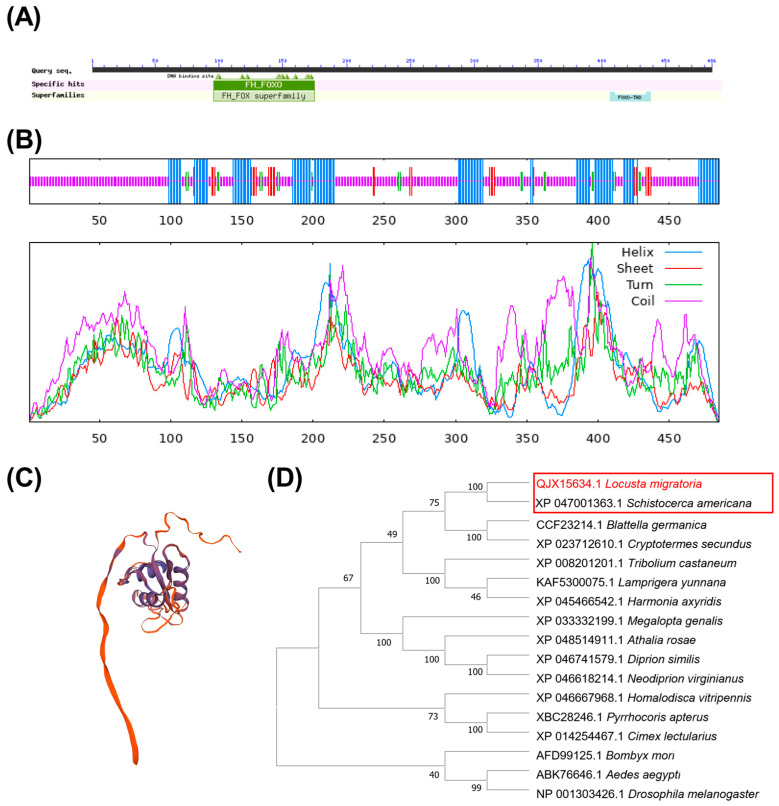
Bioinformatics analysis of *FoxO* in *L. migratoria*. (**A**) Prediction of conserved domains in LmFoxO proteins, which contain two functional domains: the FH and FoxO-TAD structure domains. (**B**) Secondary structure of LmFoxO. (**C**) Tertiary structure of *LmFoxO*. (**D**) Evolutionary tree analysis of LmFoxO using the neighbor-joining method with insect FoxO protein sequences from *S. americana*, *B. germanica*, *C. secundus*, *H. vitripennis*, *T*. *castaneum*, *H*. *axyridis*, *C*. *lectularius*, *L*. *yunnana*, *M*. *genalis*, *P*. *apterus*, *A*. *rosae*, *D*. *similis*, *N. virginianus*, *D*. *melanogaster*, *B*. *mori*, and *A*. *aegypti*.

The cDNA sequence of *LmFoxO* (GenBank accession number QJX15634.1) was identified based on the transcriptome data. The predicted protein has a calculated molecular mass (MM) of approximately 52,186 and an isoelectric point (pI) of 9.30, as determined using the ExPASy Proteomics website. The amino acid sequence of FoxO consists of an FH domain spanning residues 95–175 and a FoxO-TAD domain spanning residues 406–438 (Figure 1A). The secondary structure analysis revealed that the FoxO protein comprises α-helices, extended chains, β-turns, and random coils (Figure 1B), with random coils constituting the largest proportion at 63.71%, which is consistent with the predicted tertiary structure (Figure 1C).

The similarity of locusts with other species was assessed using a BLAST search on the NCBI website, and the top 10 sequences with the highest identity were selected. The multiple sequence results obtained from MEGA 11 were utilized to construct the evolutionary tree. A significant level of homology was revealed in the amino acid sequence of FoxO between *L. migratoria* and *Schistocerca americana* (XP_047001363.1) (Figure 1D).

### 3.2. Tissue-Specific Expressions of FoxO and Key Hippo-Related Genes in L. migratoria

**Figure 2:**
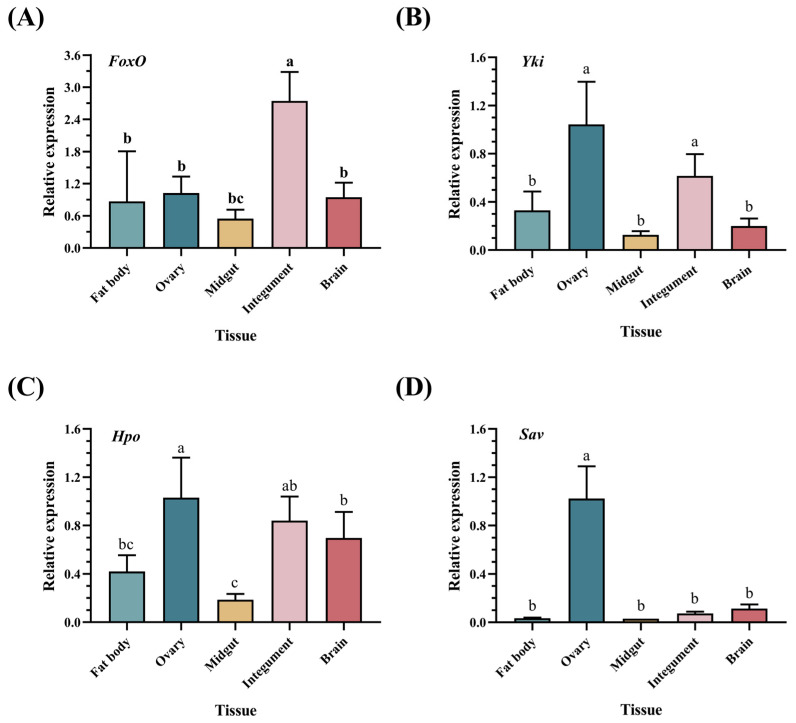
Relative expression of *FoxO* in different tissues. The tissue-specific expression patterns of *FoxO* and Hippo-related genes in *L. migratoria*, including (**A**) *FoxO*, (**B**) *Yki*, (**C**) *Hpo*, and (**D**) *Sav* in the fat bodies, ovaries, midguts, integuments, and brains of female adults within 12 h post-eclosion. The values are presented as means ± SEs (*n* = 3). Different letters indicate significant differences among the tissues (*p* < 0.05) based on one-way ANOVA. Three biological replicates were established for each developmental stage, with no fewer than five test worms.

To investigate the tissue-specific expression patterns of *FoxO* and the key genes of the Hippo pathway, we performed RT-qPCR to detect the transcript levels in five tissues from female locusts. *FoxO* expression was detected in all five tissues, with predominant expression in the integument and relatively high expression in the ovary (Figure 2A). *Yki* exhibited prominent expression in the ovary and relatively high expression in the integument (Figure 2B). *Hpo* showed relatively higher expression levels in the ovary compared to the integument, and the lowest expression levels were observed in the midgut (Figure 2C). *Sav* displayed significantly higher expression levels in the ovary compared to the other tissues (Figure 2D). These findings suggest that *LmFoxO* and the Hippo pathway may play a role in the reproductive processes of *L. migratoria*.

### 3.3. Effects of dsFoxO on Expressions of FoxO and Key Hippo-Related Genes

**Figure 3:**
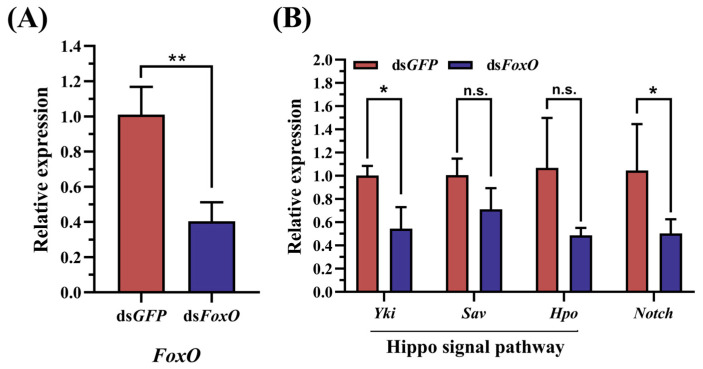
Effect of RNAi on the relative expressions of *FoxO* and Hippo-related genes in *L. migratoria*. (**A**) Changes in relative expressions of *FoxO* genes following RNAi treatment. Impact of *FoxO* RNAi injection on the expressions of (**B**) Hippo-related genes and *Notch*. The control group received an equal injection volume of ds*GFP*. Values are presented as means ± standard errors (SEs). * Denotes a significant difference between the two groups using Student’s *t*-test (* *p* < 0.05 and ** *p* < 0.01), with three biological replicates consisting of no less than five test insects per treatment.

*FoxO* RNAi resulted in a significant 59.75% reduction in the transcript abundance of *FoxO* in the fat body of adult females at 5 days after the treatment (Figure 3A). To investigate the impact of *LmFoxO* interference on the key genes involved in the Hippo pathway, we assessed the expression levels of three crucial genes using RT-qPCR. The data revealed that the knockdown of *LmFoxO* effectively downregulated the *Yki* transcript levels, as well as reduced the expressions of the *Sav* and *Hpo* genes (Figure 3B), indicating that ds*FoxO* influenced the Hippo pathway and inhibited related gene expressions.

### 3.4. Effects of FoxO Silencing on L. migratoria Reproduction

**Figure 4:**
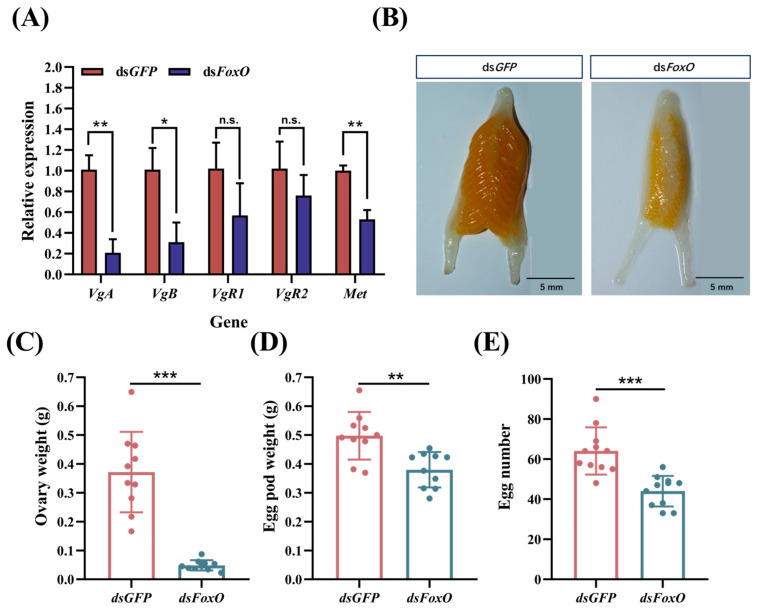
Effects of *FoxO* silencing on reproduction in *L. migratoria*. (**A**) Impact of ds*FoxO* injection on the expressions of reproduction-related genes, including *VgA*, *VgB*, *VgR1*, *VgR2*, and *Met*. The values are presented as means ± SEs. Changes in the (**B**) ovarian morphology, (**C**) ovary weight, (**D**) egg pod weight, and (**E**) egg number after *FoxO* silencing were assessed. Statistical significance was determined using Student’s *t*-test (* *p* < 0.05, ** *p* < 0.01, and *** *p* < 0.001). Each treatment consisted of three biological replicates with no less than five test insects per replicate. The scale bar represents 5 mm.

To investigate the impact of *FoxO* silencing on locust reproduction, we examined the expression levels of the reproduction-related genes as well as the ovarian development. Our findings revealed a significant downregulation in the mRNA levels of *VgA*, *VgB*, and *Met* in the fat body (Figure 4A), indicating the inhibition of vitellogenin synthesis. The expression levels of *VgR1* and *VgR2* in the ovary were also observed to be downregulated, although there was no significant difference. Furthermore, there was a notable reduction in ovarian weight and severe atrophy in ovarian development (Figure 4B,C). Additionally, we observed substantial decreases in both the egg pod weight and number following the ds*FoxO* injection (Figure 4D,E). These results underscore the profound impact of *FoxO* silencing on *L. migratoria*’s reproductive capabilities.

### 3.5. Effects of FoxO Silencing on Glycogen and Trehalose in L. migratoria

**Figure 5:**
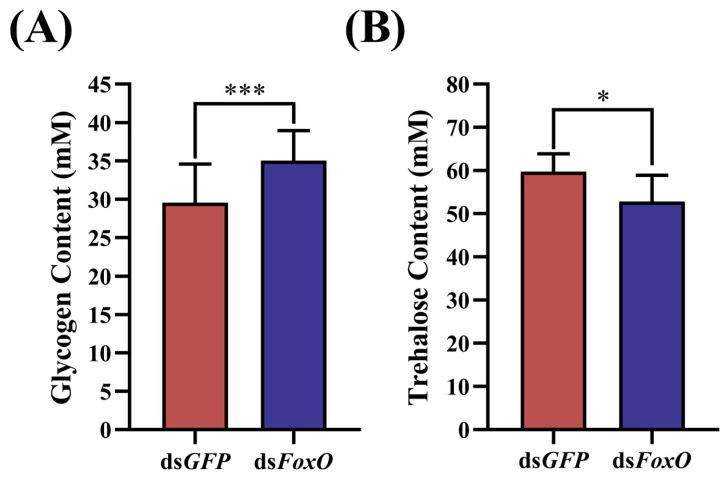
Effects of *FoxO* silencing on (**A**) glycogen and (**B**) trehalose in *L. migratoria*. The control group was injected with ds*GFP*. Values are presented as means ± SEs. * Denotes a significant difference between the two groups using Student’s *t*-test (* *p* < 0.05 and *** *p* < 0.001), with three biological replicates consisting of no less than five test insects per treatment.

The active reproduction of insects is closely intertwined with their metabolism. Therefore, we aimed to explore whether *FoxO* contributes to the reproduction–metabolism balance in locusts. We measured the trehalose and glycogen levels of females under different experimental conditions. Upon ds*FoxO* injection, the locusts exhibited significantly increased glycogen contents but significantly decreased trehalose contents, indicating a regulatory role for *FoxO* (Figure 5).

## 4. Discussion

The Forkhead box (Fox) protein family, consisting of 19 subfamilies, is a widely distributed transcription factor family in animals that is characterized by a conserved DNA-binding domain (the Forkhead-box or Fox) [38,39]. Among these subfamilies, *FoxO* has been extensively studied and exhibits a highly conserved structure and function across species [14,40,41]. In this study, we identified *LmFoxO* and found that the amino acid sequence of FoxO consists of an FH domain and a FoxO-TAD domain (Figure 1A), which demonstrates the conservation of *FoxO*. The multiple sequence results obtained from MEGA 5.1 were utilized to construct the evolutionary tree. The multiple sequence alignment revealed a significant level of homology in the amino acid sequence of FoxO between *L. migratoria* and *Schistocerca americana* (XP_047001363.1) (Figure 1D).

Multiple studies have demonstrated that the Hippo signaling pathway serves as a primary target through which *FoxO* governs cellular homeostasis and lifespan regulation [26,42,43,44]. Additionally, gene ontology analysis has revealed the enrichment of differentially expressed *FoxO* target genes in aging fat bodies within the Hippo signaling pathway [45]. Previous studies employing ChIP-Seq technology have confirmed the Hippo pathway as a major target of *FoxO* in wild-type fruit flies [45]. Regulators of the Hippo pathway are among the FOXO-dependent upregulated genes [46] (Figure S1). Alternatively, via the STRING database, we predicted an interaction between FOXO proteins and the key proteins of the Hippo pathway in *Drosophila* (Figure S2; Table S1). These aforementioned investigations provide a theoretical foundation for exploring the relationship between *FoxO* and the Hippo signaling pathway, as well as their joint mechanisms that regulate insect reproduction. In female locusts with disrupted *FoxO* function, there was a significant reduction in the expressions of the key genes *Yki*, *Hpo*, and *Sav* (Figure 3). These findings confirm that the Hippo pathway is targeted by *FoxO* in *L. migratoria*.

In this study, we initially assessed the expression profiles of both *FoxO* and the key genes involved in the Hippo pathway. We observed the widespread expression of *FoxO* across various tissues in the female locusts, with predominant expression in the integument tissue and relatively high expression levels in the ovaries (Figure 2A), suggesting the potential involvement of *FoxO* in diverse biological processes, including reproduction. We selected female adult locusts that had undergone molting 12 h prior tothe tissue expression analysis. This stage is a critical period for cuticle development, as the locusts have just completed molting, and yolk formation has not yet commenced [47]. The experimental findings revealed the predominant expression of *FoxO* in the epidermal tissues, with relatively high expression levels observed in the ovaries (Figure 2A). As a downstream target gene of cellular nutrients, growth factors, and insulin signaling pathways (IIS), FoxO plays a regulatory role in physiological processes such as growth, development, and reproduction, including insect molting and metamorphosis. In *Bombyx mori*, the transcriptional levels of FOXO increase during the ecdysone hormone 20E-induced molting and pupation processes, highlighting its crucial involvement [48]. FOXO silencing in *Helicoverpa armigera* results in failed molting and the inhibition of the 20E signal gene expression, further confirming its necessity during insect molting and metamorphosis [49]. Therefore, we hypothesize that the primary function of *FoxO* in newly molted locusts lies in epidermal development rather than in ovarian development. However, the specific mechanism requires further investigation. Notably, the key genes associated with the Hippo pathway exhibited significantly higher expression levels, specifically within the female locust ovaries, compared to other tissues such as the brain, integument, and midgut (Figure 2B). Based on these findings, we predict that both *FoxO* and the Hippo signaling pathway play crucial roles in insect reproduction.

*FoxO* exerts an impact on reproduction in various insects [2,50,51]. In the mosquitos *Culex pipiens* and *Aedes aegypt*, *FoxO* knockdown represses *Vg* expression, leading to reduced reproductive rates [52,53]. The depletion of *FoxO* also suppresses *Vg* expression and diminishes ovarian development in the soybean pod borer *Maruca vitrata* [54]. Collectively, these studies support our observation that *FoxO* knockdown in vitellogenic female locusts significantly reduces *Vg* expression while impeding oocyte maturation and arresting ovarian growth (Figure 4). Following interference with the *FoxO* expression, the depletion of *FoxO* leads to a significant reduction in adipocyte polyploidy, accompanied by decreased *Vg* expression and impaired oocyte maturation, resulting in hindered ovarian growth in locusts [16]. Wu et al. provide evidence that *FoxO* is a crucial player in JH-dependent polyploidization, vitellogenesis, and egg development, which extends the view of JH action in insect cell polyploidization and vitellogenesis; however, the regulatory role of *FoxO* in insect vitellogenesis is not well defined.

*Notch* plays a crucial role in insect oogenesis [29,55,56]. The loss of function of *Notch* arrests the development of stalk and polar cells [57]. In *L. migratoria*, Notch-depleted adult females had blocked oocyte maturation and arrested ovarian growth [10]. This is consistent with our findings. In our study, we demonstrated that the ds*FoxO* treatment resulted in significantly decreased *Notch* expression levels (Figure 3B), accompanied by reduced Vg transcripts (Figure 4A), arrested oocyte maturation, and blocked ovarian growth (Figure 4B). Additionally, the Hippo pathway plays a crucial role in regulating the Notch receptor levels in follicle cells [29,30,58]. In *D. melanogaster*, the control of the mitosis–endocycle switch in follicular cells has been associated with the Notch pathway, as Notch signaling is attenuated in Hippo mutants [30,59]. In *Drosophila* imaginal discs, the Hippo pathway regulates membrane receptor trafficking, including the Notch receptor [60]. Our study demonstrated that the ds*FoxO* treatment resulted in significantly decreased levels of the key genes of Hippo (Figure 3B), accompanied by reduced *Notch* transcripts (Figure 3B) and suppressed reproduction (Figure 4). In the previous section, we demonstrated that the Hippo signaling pathway is one of the targets of *FoxO* in *L. migratoria* and that it promotes Notch signaling in the regulation of cell differentiation and proliferation, and oocyte polarity. Although we could not exclude the involvement of other potential signaling molecules, the findings in the present study, together with our previous analysis, suggest that *FoxO* regulates locust reproduction through Hippo–*Notch*.

Egg production is one of the most energy-demanding events in the adult lives of female insects. In addition to Vg, large amounts of carbohydrates and lipids are required to meet the energy demands of oocyte growth [61]. The insulin signaling pathway is involved in the regulation of the circulating sugar levels; thus, *FoxO* plays an important role in the regulation of sugar levels as a downstream target gene of the insulin signaling pathway [62]. It is obvious that the female reproductive processes require considerable amounts of energy-rich substrates and *FoxO*-mediated energy mobilization may be involved in the regulation of egg production [63,64]. In insects, trehalose accumulation primarily arises from glycogen breakdown metabolism [65,66,67]. The change pattern of the trehalose content is opposite to that of the glycogen content, which aligns with the experimental results obtained in this study. Our experiment revealed a significant decrease in the trehalose content (Figure 5B) after *FoxO* RNAi, while the glycogen content increased significantly (Figure 5A). Decreasing glycogen storage leads to a metabolic shift, resulting in increased internal trehalose [68]. Considering the dynamic fluctuations in the total sugar and glycogen contents, a reciprocal conversion between trehalose and glycogen may occur. Trehalose homeostasis regulates vitellogenesis and oocyte development in female insects. In *L. migratoria* and *P. americanahe*, trehalose involvement in Vg synthesis in the fat body and Vg uptake by the developing oocytes have been confirmed [69,70]. In our experiments, *FoxO* interference severely reduced the trehalose content, thus greatly reducing the synthesis and uptake of Vg in locusts disrupted by *FoxO.* This also demonstrates that *FoxO*-mediated energy mobilization is involved in the regulation of egg production.

## 5. Conclusions

Our study provides evidence that *FoxO* promotes fat body vitellogenesis in locusts through the Hippo signaling pathway–*Notch*. *FoxO* silencing results in decreased female locust reproduction. In addition, *FoxO*-mediated energy mobilization is involved in the regulation of egg production. These findings expand our understanding of the physiological functions of *FoxO* in insects and emphasize its significance in locust reproduction. Overall, these results highlight the potential of targeting *FoxO* as a novel molecular approach for controlling *L. migratoria*.

## Figures and Tables

**Table 1:** Primers for PCR.

Primer Name	F-Primer Sequence [5′–3′]	R-Primer Sequence [5′–3′]	Method
*FoxO1*	AGATGGACCCGTCGTTCGAG	GGCTGAAGTCTGAAGTTGAAGTC	cDNA Clones
*FoxO2*	CTGGACGTGGTGGTGAAGCA	CGTGCTTGATCACCTCGTCC	
*FoxO3*	GCCAAGAAGAACACCAGCC	CGTCTCGATGTTGAGGTTGAGG	
*GFP*	AAGGGCGAGGAGCTGTTCACCG	CAGCAGGACCATGTGATCGCGC	
*FoxO*	GAACTCGATCCGGCATAACC	CGCCTCCACCTTCTTCTTG	RT-qPCR
*VgA*	CCCACAAGAAGCACAGAACG	TTGGTCGCCATCAACAGAAG	
*VgB*	GCACTTAGCAGCATTAAGACCC	GGCAACGATAGATGGATAGGAC	
*VgR1*	ATAAAGGTCTACCATCCAGCCC	GACAGGCACAGGTGTAGGAGTT	
*VgR2*	GGCAAAAGGGATCACTCGA	GCCACCATCAGCCCAAAAT	
*Met*	GCGGTCACCTCTTGTCAATAAT	CACTTTCTGATGCTGCCCTAA	
*Hpo*	GCTGAAAACATAAAGGGAGG	CTGGAATGGATTCGGAGG	
*Sav*	CTGCTTTGGTTCCTTCAGT	GTTGGTAGCCCTTCTTTCTC	
*Yki*	AAGCCCCTGCTCGTATTTAT	TCTATCCGCACCACCAAGTT	
*Notch*	CGGAAACCGAGTGTCAAG	CGGGCTGGGAATGCTA	
ds*FoxO*	TAATACGACTCACTATAGGGAGATGGACCCGTCGTTCGAG	TAATACGACTCACTATAGGGGGCTGAAGTCTGAAGTTGAAGTC	dsRNA Synthesis
ds*GFP*	TAATACGACTCACTATAGGGAAGGGCGAGGAGCTGTTCACCG	TAATACGACTCACTATAGGGCAGCAGGACCATGTGATCGCGC	

## Data Availability

The data will be made available upon request.

## Supplementary Materials

The following supporting information can be downloaded at https://www.mdpi.com/article/10.3390/insects15110891/s1, Supplementary File S1: The cDNA sequence and primers for *FoxO* clones and sequencing results; Figure S1: Heatmap depicting the increases in mRNA expression [46]; Figure S2: Interaction between FOXO protein and key proteins in Hippo pathway in *Drosophila melanogaster*; Table S1: Interactions between FoxO and Hpo, Sd, and Yki in *Drosophila melanogaster*.
