# Supplementary material for: RNAi-Mediated FoxO Silencing Inhibits Reproduction in Locusta migratoria
Source: Insects. 2024 Nov 14;15(11):891. doi: 10.3390/insects15110891 (PMC11594837; doi:10.3390/insects15110891)
Supplement: Supplementary file 1 [file insects-15-00891-s001.zip › Figure S1. Heatmap depicting the increases in mRNA expression..pdf]

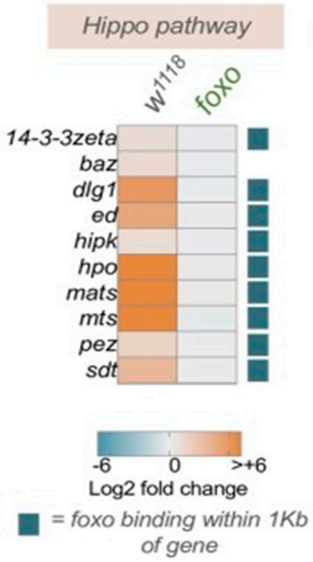

Fig. S1. Heatmap depicting the increases in mRNA expression. Log2-fold change, hypoxia vs normoxia) of Hippo pathway genes in w1118 and foxo mutants. Blue squares indicate genes previously shown to have FOXO binding within 1 kb of the gene as measured by ChIP (*Ding et al., 2022*).
