# Supplementary material for: RNAi-Mediated FoxO Silencing Inhibits Reproduction in Locusta migratoria
Source: Insects. 2024 Nov 14;15(11):891. doi: 10.3390/insects15110891 (PMC11594837; doi:10.3390/insects15110891)
Supplement: Supplementary file 1 [file insects-15-00891-s001.zip › Figure S2. Interaction between FOXO protein and key proteins in Hippo pathway in Drosophila melanogaster..pdf]

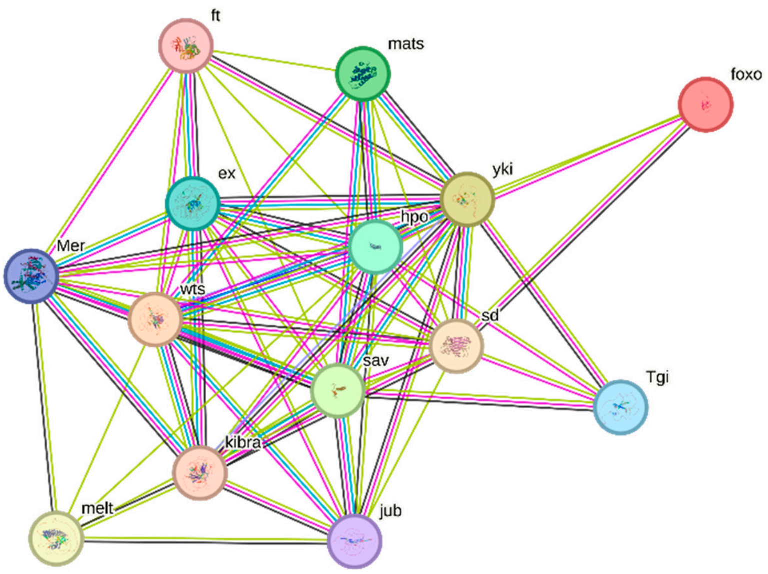

Fig. S2. Interaction between FOXO protein and key proteins in Hippo pathway in *Drosophila melanogaster*. foxo: Forkhead box protein O; **yki:** Transcriptional coactivator yorkie; **hpo:** Serine/threonine-protein kinase hippo; **mats:** MOB kinase activator-like 1; **sd:** Scalloped, isoform Q; **sav:** Scaffold protein Salvador; **wts:** Serine/threonine-protein kinase Warts; **Mer:** Moesin/ezrin/radixin homolog 2; **Tgi:** Tondu-domain-containing Growth Inhibitor; **ft:** Cadherin-related tumor suppressor; **ex:** Protein expanded, Regulator of the Hippo/SWH (Sav/Wts/Hpo) signaling pathway; **jub:** LIM domain-containing protein jub; **kibra:** Protein kibra, Regulator of the Hippo/SWH (Sav/Wts/Hpo) signaling pathway; **melt:** Protein melted, participates in fat metabolism regulation by recruiting FOXO and the TSC1-TSC2 complex to the cell membrane. Yki, Sd, Hpo, Mats, Sav, Wts and Mer belong to the Hippo signaling pathway.
