# Supplementary material for: RNAi-Mediated FoxO Silencing Inhibits Reproduction in Locusta migratoria
Source: Insects. 2024 Nov 14;15(11):891. doi: 10.3390/insects15110891 (PMC11594837; doi:10.3390/insects15110891)
Supplement: Supplementary file 1 [file insects-15-00891-s001.zip › Supplementary file S1.pdf]

The cDNA sequence and primers for *FoxO* clones and sequencing results

>MN427928.1 Locusta migratoria FoxO mRNA, partial cds

ATGATGGAGCAGGGCCCGCCCG**AGATGGACCCGTCGTTCGAG**CCGCAG

CAGCGCGCGCGCTCCAACACGTGGCCGCTGCCGCGCCCCGACAACTTC**CT**

GGACGTGGTGGTGAAGCAGGAGCCGGGCGCGGAGGCGGGGCCGGCGGC

CGGCATGCTGGGCCCGTGCGCCGCGGCCGTGCACGGCGCGGCCGGCTCGC

CCGTCGGCGCCGCCGGAGAGCCCGTCTACCTGTCGCCCGCCGCCGTCGTC

GGCGCC**GCCAAGAAGAACACCAGCC**GCCGCAACGCCTGGGGCAACCTG

TCCTACGCCGACCTCATCACGCAGGCCATCCAGTCGGCGCCCGACCAGCG

CCTCACGCTGTCGCAGATATACGAGTGGATGGTGCAGAACGTGCCCTACT

TCAAGGACAAGGGCGACAGCAACAGCTCCGCCGGGTGGAAGAACTCGAT

CCGGCATAACCTGTCGCTGCACAATCGGTTCATGCGCGTGCAGAACGAGG

GCACCGGCAAGTCCAGCTGGTGGATGATCAACCCGGACGCCAAGCCCGGC

AAGAGCGCGCGCCGTCGCGCCACCTCCATGGAGACCTCCAAGTTCGAGAA

GCGCCGCGGCCGCGTCAAGAAGAAGGTGGAGGCGCTGCGCAACGGGCTG

TCGGCGGCGGACGCGACGCCGTCGCCGTCCAGCTCGGTGTCGGAGGGCCT

GGACCTGTTCCCCGACTCGCCGCTGCACGCCGGCTTCCAGCTGAGCCCCG

ACTTCCGGCCCCGCGCCTCCAGCAACGCGTCGTCGTGCGGCGGCCGGCTG

TCGCCCATCCCGTCCGTGCTGGGCATGGAGCACGAGTGGGGCCCGCAGCC

GCCCCCGCAGCAGCCGCCCCCACAGCAGCCGCCCCCGCAGCAGGCGCCGC

CCCCGCAGTACGGGCTGGAGCAGCTGGCGGGCAACCTAGCCGAGAGCAT

GAAGCTGCACCACGAGACGGCGCAGCCGGCCTACCTGTTCCAGCACGCGC

CGCCGCCGCCCTACCAGCCGCAGCCGCCGCCCCCGCCGCCGCCCTACATG

GCCGGCGCCGGGCCCTACGCCGCCTGCCGCCTCTCGCCGTCGTACGAGCC

CGAGTCGCCGCCCACGCCGCAGCCGCAGCCGCCGCCGCCGCCGCCCCCGC

AGCCCAGCGGGCCCTCCACCATGATGGGCCAGCTGATGGGCGCGCTCAAC

AACTCGACGCTGCTCGACGA**CCTCAACCTCAACATCGAGACG**CTGCACG

GCGGCTTCGACTGCAACGT**GGACGAGGTGATCAAGCACG**AGCTGAGCA

TGGACGGCAGCCTC**GACTTCAACTTCAGACTTCAGCC**AGCACCACCACC

ACCAGGCGATGGTGGCGGGCGGCGGCGCCATGGCGGCCGCGGACGGCGT

GCACCACCAGCAGCACGCGGCCGCGCAGGGCGCCGCGCCCTACGCCACC

GGGCCCTCCTGGGTGCACTAGCCA

Primers for *FoxO* clones

FoxO 1

F1: 5-AGATGGACCCGTCGTTCGAG-3 （23-42）

R1: 5-GGCTGAAGTCTGAAGTTGAAGTC-3 （1303-1325）

FoxO 2

F2: 5-CTGGACGTGGTGGTGAAGCA-3 （97-116）

R2: 5-CGTGCTTGATCACCTCGTCC-3 （1260-1279）

FoxO 3

F3: 5-GCCAAGAAGAACACCAGCC-3 （253-271）

R3: 5-CGTCTCGATGTTGAGGTTGAGG-3 （1212-1233）

Notes: Due to the high GC content in the *FoxO* genome, nested PCR was chosen for its amplification. Here we designed three pairs of primers for nested PCR, named *FoxO 1*, *FoxO 2* and *FoxO 3*.

Sequencing results

>FoxO

GGGGCCCTGCCAGTCGACGATTGCCAAGAGACACCAGCCGCCGCAACGCCTGGGGCA

ACCTGTCCTACGCAGACCTCATCACGCAGGCCATCCAGTCGGCGCCCGACCAGCGCCT

CACGCTGTCGCAGATATACGAGTGGATGGTGCAGAACGTGCCCTACTTCAAGGACAAG

GGCGACAGCAACAGCTCCGCCGGGTGGAAGAACTCGATCCGGCATAACCTGTCGCTG

CACAATCGGTTCATGCGCGTGCAGAACGAGGGCACCGGCAAGTCCAGCTGGTGGATG

ATCAACCCGGACGCCAAGCCCGGCAAGAGCGCGCGCCGTCGCGCCACCTCCATGGAG

ACCTCCAAGTTCGAGAAGCGCCGCGGCCGCGTCAAGAAGAAGGTGGAGGCGCTGCG

CAACGGGCTGTCGGCGGCGGACGCGACGCGGTCGCCGTCCAGCTCGGTGTCGGAGGG

CCTGGACCTGTTCCCCGACTCGCCGCTGCACGCCGGCTTCCAGCTAAGCCCCGACTTC

CGGCCCCGCGCCTCCAGCAACGCGTCGTCGTGCGGCGGCCGGCTGTCGCCCATCCCGT

CCGTGCTGGGCATGGAGCACGAGTGGGGCCCGCAGCCGCCCCCGCAGCACCGCACCC

CACAGCAG

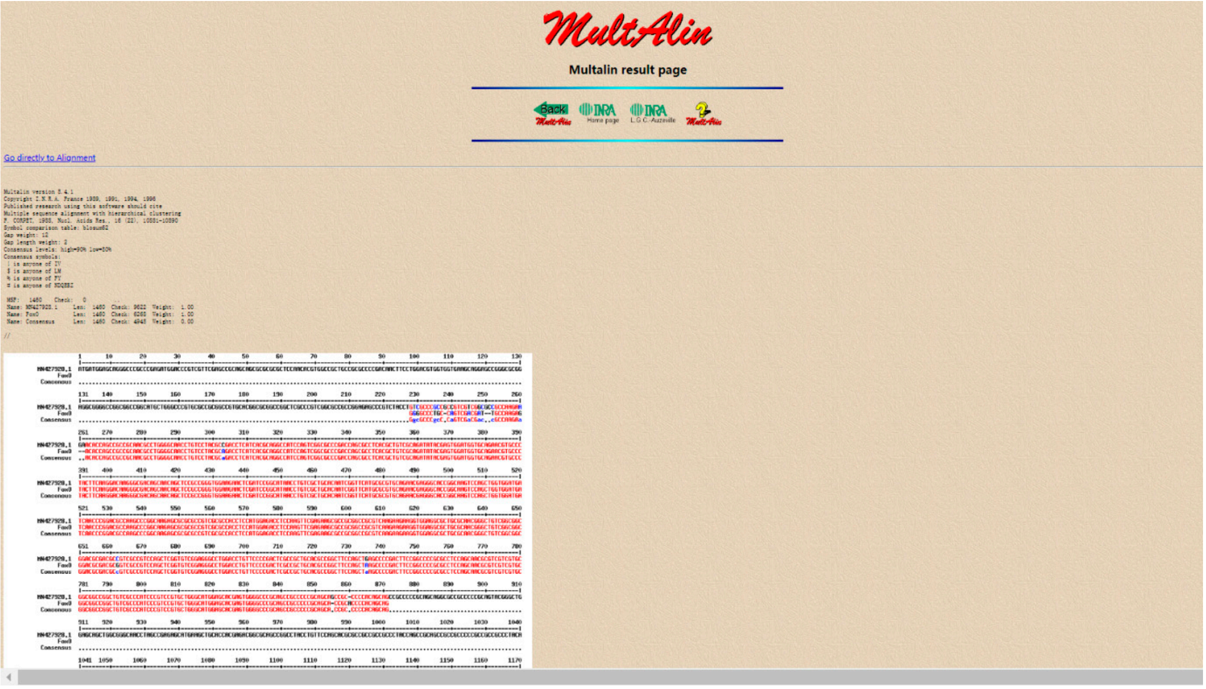

Multalin version 5.4.1

Copyright I.N.R.A. France 1989, 1991, 1994, 1996

Published research using this software should cite

Multiple sequence alignment with hierarchical clustering

F. CORPET, 1988, Nucl. Acids Res., 16 (22), 10881-10890

Symbol comparison table: blosum62

Gap weight: 12

Gap length weight: 2

Consensus levels: high=90% low=50%

Consensus symbols:

! is anyone of IV

$ is anyone of LM

% is anyone of FY

is anyone of NDQEBZ

MSF: 1460 Check: 0 ..

Name: MN427928.1 Len: 1460 Check: 9622 Weight: 1.00

Name: FoxO Len: 1460 Check: 6268 Weight: 1.00

Name: Consensus Len: 1460 Check: 4945 Weight: 0.00

//

1 50

MN427928.1 ATGATGGAGC AGGGCCCGCC CGAGATGGAC CCGTCGTTCG AGCCGCAGCA

FoxO ....

Consensus ....

51 100

MN427928.1 GCGCGCGCGC TCCAACACGT GGCCGCTGCC GCGCCCCGAC AACTTCCTGG

FoxO ....

Consensus ....

101 150

MN427928.1 ACGTGGTGGT GAAGCAGGAG CCGGGCGCGG AGGCGGGGCC GGCGGCCGGC

FoxO ....

Consensus ....

151 200

MN427928.1 ATGCTGGGCC CGTGCGCCGC GGCCGTGCAC GGCGCGGCCG GCTCGCCCGT

FoxO ....

Consensus ....

201 250

MN427928.1 CGGCGCCGCC GGAGAGCCCG TCTACCTGTC GCCCGCCGCC GTCGTCGGCG

FoxO ....GGG GCCCTGC.CA GTCGACGAT.

Consensus ....Ggc GCCCgcC.Ca GTCGaCGac.

251 300

MN427928.1 CCGCCAAGAA GAACACCAGC CGCCGCAACG CCTGGGGCAA CCTGTCCTAC

FoxO .TGCCAAGAG ..ACACCAGC CGCCGCAACG CCTGGGGCAA CCTGTCCTAC

Consensus .cGCCAAGAa ..ACACCAGC CGCCGCAACG CCTGGGGCAA CCTGTCCTAC

301 350

MN427928.1 GCCGACCTCA TCACGCAGGC CATCCAGTCG GCGCCCGACC AGCGCCTCAC

FoxO GCAGACCTCA TCACGCAGGC CATCCAGTCG GCGCCCGACC AGCGCCTCAC

Consensus GCaGACCTCA TCACGCAGGC CATCCAGTCG GCGCCCGACC AGCGCCTCAC

351 400

MN427928.1 GCTGTCGCAG ATATACGAGT GGATGGTGCA GAACGTGCCC TACTTCAAGG

FoxO GCTGTCGCAG ATATACGAGT GGATGGTGCA GAACGTGCCC TACTTCAAGG

Consensus GCTGTCGCAG ATATACGAGT GGATGGTGCA GAACGTGCCC

TACTTCAAGG

401 450

MN427928.1 ACAAGGGCGA CAGCAACAGC TCCGCCGGGT GGAAGAACTC GATCCGGCAT

FoxO ACAAGGGCGA CAGCAACAGC TCCGCCGGGT GGAAGAACTC GATCCGGCAT

Consensus ACAAGGGCGA CAGCAACAGC TCCGCCGGGT GGAAGAACTC GATCCGGCAT

451 500

MN427928.1 AACCTGTCGC TGCACAATCG GTTCATGCGC GTGCAGAACG AGGGCACCGG

FoxO AACCTGTCGC TGCACAATCG GTTCATGCGC GTGCAGAACG AGGGCACCGG

Consensus AACCTGTCGC TGCACAATCG GTTCATGCGC GTGCAGAACG AGGGCACCGG

501 550

MN427928.1 CAAGTCCAGC TGGTGGATGA TCAACCCGGA CGCCAAGCCC GGCAAGAGCG

FoxO CAAGTCCAGC TGGTGGATGA TCAACCCGGA CGCCAAGCCC GGCAAGAGCG

Consensus CAAGTCCAGC TGGTGGATGA TCAACCCGGA CGCCAAGCCC GGCAAGAGCG

551 600

MN427928.1 CGCGCCGTCG CGCCACCTCC ATGGAGACCT CCAAGTTCGA GAAGCGCCGC

FoxO CGCGCCGTCG CGCCACCTCC ATGGAGACCT CCAAGTTCGA

GAAGCGCCGC

Consensus CGCGCCGTCG CGCCACCTCC ATGGAGACCT CCAAGTTCGA GAAGCGCCGC

601 650

MN427928.1 GGCCGCGTCA AGAAGAAGGT GGAGGCGCTG CGCAACGGGC TGTCGGCGGC

FoxO GGCCGCGTCA AGAAGAAGGT GGAGGCGCTG CGCAACGGGC TGTCGGCGGC

Consensus GGCCGCGTCA AGAAGAAGGT GGAGGCGCTG CGCAACGGGC TGTCGGCGGC

651 700

MN427928.1 GGACGCGACG CCGTCGCCGT CCAGCTCGGT GTCGGAGGGC CTGGACCTGT

FoxO GGACGCGACG CGGTCGCCGT CCAGCTCGGT GTCGGAGGGC CTGGACCTGT

Consensus GGACGCGACG CcGTCGCCGT CCAGCTCGGT GTCGGAGGGC CTGGACCTGT

701 750

MN427928.1 TCCCCGACTC GCCGCTGCAC GCCGGCTTCC AGCTGAGCCC CGACTTCCGG

FoxO TCCCCGACTC GCCGCTGCAC GCCGGCTTCC AGCTAAGCCC CGACTTCCGG

Consensus TCCCCGACTC GCCGCTGCAC GCCGGCTTCC AGCTaAGCCC CGACTTCCGG

751 800

MN427928.1 CCCCGCGCCT CCAGCAACGC GTCGTCGTGC GGCGGCCGGC

TGTCGCCCAT

FoxO CCCCGCGCCT CCAGCAACGC GTCGTCGTGC GGCGGCCGGC TGTCGCCCAT

Consensus CCCCGCGCCT CCAGCAACGC GTCGTCGTGC GGCGGCCGGC TGTCGCCCAT

801 850

MN427928.1 CCCGTCCGTG CTGGGCATGG AGCACGAGTG GGGCCCGCAG CCGCCCCCGC

FoxO CCCGTCCGTG CTGGGCATGG AGCACGAGTG GGGCCCGCAG CCGCCCCCGC

Consensus CCCGTCCGTG CTGGGCATGG AGCACGAGTG GGGCCCGCAG CCGCCCCCGC

851 900

MN427928.1 AGCAGCCGC. CCCCACAGCA GCCGCCCCCG CAGCAGGCGC CGCCCCCGCA

FoxO AGCA.CCGCA CCCCACAGCA G......... ....

Consensus AGCA.CCGC. CCCCACAGCA G......... ....

901 950

MN427928.1 GTACGGGCTG GAGCAGCTGG CGGGCAACCT AGCCGAGAGC ATGAAGCTGC

FoxO ....

Consensus ....

951 1000

MN427928.1 ACCACGAGAC GGCGCAGCCG GCCTACCTGT TCCAGCACGC GCCGCCGCCG

FoxO ....

Consensus ....

1001 1050

MN427928.1 CCCTACCAGC CGCAGCCGCC GCCCCCGCCG CCGCCCTACA TGGCCGGCGC

FoxO ....

Consensus ....

1051 1100

MN427928.1 CGGGCCCTAC GCCGCCTGCC GCCTCTCGCC GTCGTACGAG CCCGAGTCGC

FoxO ....

Consensus ....

1101 1150

MN427928.1 CGCCCACGCC GCAGCCGCAG CCGCCGCCGC CGCCGCCCCC GCAGCCCAGC

FoxO ....

Consensus ....

1151 1200

MN427928.1 GGGCCCTCCA CCATGATGGG CCAGCTGATG GGCGCGCTCA ACAACTCGAC

FoxO ....

Consensus ....

1201 1250

MN427928.1 GCTGCTCGAC GACCTCAACC TCAACATCGA GACGCTGCAC GGCGGCTTCG

FoxO ....

Consensus ....

1251 1300

MN427928.1 ACTGCAACGT GGACGAGGTG ATCAAGCACG AGCTGAGCAT GGACGGCAGC

FoxO ....

Consensus ....

1301 1350

MN427928.1 CTCGACTTCA ACTTCAGACT TCAGCCAGCA CCACCACCAC CAGGCGATGG

FoxO ....

Consensus ....

1351 1400

MN427928.1 TGGCGGGCGG CGGCGCCATG GCGGCCGCGG ACGGCGTGCA CCACCAGCAG

FoxO ....

Consensus ....

1401 1450

MN427928.1 CACGCGGCCG CGCAGGGCGC CGCGCCCTAC GCCACCGGGC CCTCCTGGGT

FoxO ....

Consensus ....

1451 1460

MN427928.1 GCACTAGCCA

FoxO ....

Consensus ....
