# Supplementary material for: RNAi-Mediated FoxO Silencing Inhibits Reproduction in Locusta migratoria
Source: Insects. 2024 Nov 14;15(11):891. doi: 10.3390/insects15110891 (PMC11594837; doi:10.3390/insects15110891)
Supplement: Supplementary file 1 [file insects-15-00891-s001.zip › Table S1. Ineractions between FoxO and Hpo, Sd, Yki in Drosophila melanogaster.pdf]

Table S1 Ineractions between FoxO and Hpo, Sd, Yki in *Drosophila melanogaster*

Node1	Node2	Node1 accession	Node2 accession	score
FoxO	Hpo	*FBpp0293589*	*FBpp0304253*	0.432
	Sd		*FBpp0301994*	0.482
	Yki		*FBpp0402906*	0.467
